# PI3K signaling in the locus coeruleus: a new molecular pathway for ADHD research

**DOI:** 10.15252/emmm.201505266

**Published:** 2015-04-29

**Authors:** Emmanuel Darcq, Brigitte L Kieffer

**Affiliations:** Department of Psychiatry, Faculty of Medicine, Douglas Hospital Research Center, McGill UniversityMontreal, QC, Canada

## Abstract

Attention-deficit/hyperactivity disorder (ADHD) is a developmental disorder characterized by hyperactivity, inattention, and impulsive behaviors and has significant societal impact. ADHD is recognized as a heterogeneous disease, and genetic and/or environmental factors underlying pathogenesis remain largely unknown. There is an obvious need to increase knowledge on molecular signaling and brain pathways underlying disease development, and genetic mouse models are key to this goal. In this issue of *EMBO Molecular Medicine*, D'Andrea *et al* (2015) combine state-of-the-art genetic and behavioral approaches in the mouse to demonstrate an essential role for PI3Kγ and cAMP homeostasis in ADHD-related behaviors, through signaling mechanisms operating at the level of the locus coeruleus, the main source of noradrenaline in the brain. Furthermore, the study posits PI3Kγ knockout mice as a novel tool of high interest for modeling ADHD endophenotypes.

See also: **I D'Andrea *et al*** (July 2015)

Attention-deficit/hyperactivity disorder is one of the most common childhood disorders, affecting around 7% of children in the world (Thomas *et al*, 2015) and this disorder often persists through adolescence and into adulthood. ADHD is characterized by a combination of symptoms such as inattention, impulsivity, and hyperactivity (Gawrilow *et al*, 2014). These symptoms appear with different levels of severity and can lead to adverse harmful consequences during the entire lifetime, including anxiety, depression, delinquency, and drug abuse disorders. Despite these dramatic consequences, the causative mechanisms for ADHD are complex and unclear, and molecular mechanisms underlying ADHD are still understudied. A main hypothesis, centered on monoamine neurotransmission, supposes that the complex interactions between dopamine, noradrenaline, and serotonin neurotransmitter systems are deregulated, and human studies have associated ADHD diagnosis with genes belonging to monoamine receptors and transporters (Kebir & Joober, 2011). The majority of treatments available today are psychostimulants such as methylphenidate (MPH), a dopamine/norepinephrine transporter inhibitor, and amphetamine (Gawrilow *et al*, 2014). These treatments increase levels of available dopamine and are thought to restore monoaminergic balance, which is altered during the development of ADHD. It is clear also that treatment with psychostimulants does not treat all the patients and is often associated with adverse effects including headache, insomnia, anorexia, and weight loss. As for most psychiatric illnesses, therefore, there is a need to develop better therapeutic strategies.

To date, several genetically modified mouse lines have provided clues on genes and pathways involved in the development of ADHD-related behaviors, and these studies have been reviewed recently (Leo & Gainetdinov, 2013). Dopamine transporter (DAT) knockout (KO) mice represent one of the earliest genetic mouse models for ADHD and recapitulate several ADHD-like symptoms, including spontaneous hyperactivity (Gainetdinov & Caron, 2001), impaired learning and memory, and increased impulsivity (Yamashita *et al*, 2013). Other genetic manipulations of the DAT gene confirmed the importance of dopaminergic transmission. KO mice lacking genes that are not directly involved in dopamine signaling, for example substance P receptor and thyroid hormone receptor KO mice, also present hyperactivity although the latter mice are insensitive to MPH. As another example, mice lacking the nicotinic receptor α2 subunit also show key symptoms of ADHD, involving acetylcholine transmission. Recently, a new mutant mouse line lacking the orphan G protein-coupled receptor GPR-88 was reported to recapitulate hyperactivity-related ADHD-like symptoms (Quintana *et al*, 2012), further offering novel molecular mechanisms and molecular targets for ADHD research. In sum, although dopamine regulation is clearly central to the etiology of ADHD, there is a need to broaden knowledge beyond dopaminergic pathways, explore the role of other transmitter systems, and identify brain circuits contributing to main features of ADHD-related behaviors.

In this issue of *EMBO Molecular Medicine*, D'Andrea *et al* (2015) identified PI3Kγ as a novel actor in the search for molecular bases of ADHD. This signaling enzyme plays a key role in cell survival, proliferation, migration, and adhesion (Cantley, 2002) and has been mostly investigated in the context of immune and cardiovascular functions. PI3Kγ has been barely studied in the brain; however, one report had suggested a role of PI3Kγ in the control of behavioral flexibility involving NMDA receptor long-term depression (Kim *et al*, 2011). D'Andrea and colleagues observed enriched expression of PI3Kγ in noradrenergic (NA) neurons of the locus coeruleus (LC), which gathers most NA neurons in the brain and has been involved in behavioral flexibility and attention (Aston-Jones *et al*, 1999). The authors therefore tested whether PI3Kγ signaling in the LC may control some aspects of ADHD-related behaviors.

The authors first examined PI3Kγ KO mice in a comprehensive set of behavioral tasks that cover core features of ADHD. In addition to poor attentional performance and low behavioral flexibility in a set-shifting task, mutant mice showed strong hyperactivity. Importantly, both cognitive and motor deficits were fully reversed by MPH, the clinical reference for ADHD treatment (Gawrilow *et al*, 2014). Furthermore, PI3Kγ KO mice showed impaired memory in the Morris water maze test, as well as poor social skill with conspecifics, representing other behavioral hallmarks of the ADHD phenotype. This set of data, therefore, establish PI3Kγ-deficient mice as a mouse model for ADHD with remarkable face and predictive validity, which largely compares to the best genetic mouse models reported so far (Leo & Gainetdinov, 2013). There is no doubt that PI3Kγ KO mice will prove highly valuable to study the neurobiology of ADHD, and be a useful genetic model in efforts to develop biomarkers and novel treatment strategies.

D'Andrea *et al* then focused on the LC and dissected PI3Kγ downstream signaling events that may contribute to the ADHD phenotype. PI3Kγ signaling involves two pathways, a kinase-dependent and a kinase-independent activity, the former requiring the catalytically active kinase and generation of phosphoinositide (3,4,5)-trisphosphate (PIP3) and the latter acting principally to regulate cAMP levels in a phosphodiesterase (PDE)-specific manner (Patrucco *et al*, 2004). First, the authors found that knockin mice expressing a kinase-dead (KD) form of PI3Kγ show no behavioral alteration, indicating that ADHD-related behaviors observed in PI3Kγ KO mice recruit the kinase-independent/cAMP pathway. Second, biochemical analysis of PI3Kγ in KO mice revealed modifications of the entire cAMP pathway in the LC, including increased cAMP levels via PDE4D in both cytosolic and membrane compartments of NA neurons, increased phosphorylation of the transcription factor cAMP response binding protein (CREB), as well as increased noradrenaline, and decreased dopamine in LC projection areas, including striatum and prefrontal cortex. Third, virally mediated overexpression of a dominant-negative form of CREB in the LC reversed the ADHD-like phenotypes of PI3Kγ KO mice to wild-type levels and restored NA/DA unbalance in their striatum and prefrontal cortex. Fourth, overexpression of a constitutive active form of CREB in the LC of wild-type mice induced behavioral alterations, similar to those observed in PI3Kγ KO mice, including attention-deficit, hyperactivity, and altered NA/DA in the prefrontal cortex and striatum. The authors conclude that PI3Kγ regulates ADHD-related behaviors via a kinase-independent but CREB-dependent mechanism in noradrenergic neurons of the LC, which controls NA/DA in prefrontal cortex and striatum (Fig1). Identification of this signaling pathway as a key player in the control of NA neuron activities opens attractive perspectives in ADHD research and may prove important in the broad area of developmental brain disorders.

**Figure 1:**
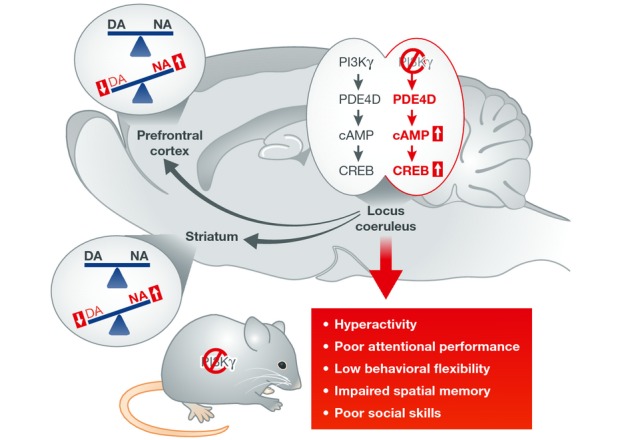
Role for PI3Kγ signaling in core symptoms of ADHD PI3Kγ is abundant in noradrenergic neurons of the locus coeruleus (LC), which constitute the main source of noradrenaline in the brain. PI3Kγ gene KO in the mouse leads to increased CREB activation via elevation of cAMP levels in the LC and alters the dopamine/noradrenaline (DA/NA) balance in projection areas (prefrontal cortex and striatum). Those modifications facilitate the development of core ADHD-related phenotypes, including hyperactivity and attention deficits, as well as secondary features such as memory and social impairments. Overexpression of CREB in the LC of normal animals produces similar behavioral changes, and down-regulation of CREB activity in the LC of mutant mice reverses the phenotype.

In the future, investigation of PI3Kγ human gene variants in relation to ADHD will certainly be noteworthy. Also, as neuroimaging methods progress in animal research, it will be interesting to determine whether PI3Kγ-mediated regulation of LC neuron activities impact brain connectivity patterns, in a manner that could be reminiscent to whole-brain alterations observed in some ADHD patients (Konrad & Eickhoff, 2010). Finally, the PI3Kγ KO mouse line will serve as an excellent ADHD model to develop and test novel therapeutic strategies, and represents a true progress in a field where animal models are particularly scarce.
